# The Effect of Schooling on Basic Cognition in Selected Nordic Countries

**DOI:** 10.5964/ejop.v13i4.1339

**Published:** 2017-11-30

**Authors:** Bert Jonsson, Maria Waling, Anna S. Olafsdottir, Hanna Lagström, Hege Wergedahl, Cecilia Olsson, Eldbjørg Fossgard, Asle Holthe, Sanna Talvia, Ingibjorg Gunnarsdottir, Agneta Hörnell

**Affiliations:** a Department of Psychology, Umeå University, Umeå, Sweden; b Department of Food and Nutrition, Umeå University, Umeå, Sweden; c School of Education, University of Iceland, Reykjavik, Iceland; d Turku Institute of Child and Youth Research, University of Turku, Turku, Finland; e Faculty of Education, Western Norway University of Applied Sciences, Bergen, Norway; f Unit for Nutrition Research, Landspitali – The National University Hospital of Iceland and Faculty of Food Science and Nutrition, School of Health Sciences, University of Iceland, Reykjavík, Iceland; Webster University Geneva, Geneva, Switzerland; The Maria Grzegorzewska University, Warsaw, Poland

**Keywords:** schooling effects, cognitive functioning, working memory, processing speed, attention, response control, inhibition

## Abstract

The present study investigated schooling effects on cognition. Cognitive data were collected as part of a research project (ProMeal) that investigated school meals and measured the intake of school lunch in relation to children’s health, cognitive function, and classroom learning in four Nordic countries, among children between 10–11 years of age. It was found that Finnish pupils attending 4th grade were not, on any measure, outperformed by Norwegian and Icelandic pupils attending 5th and Swedish pupils attending 4th grade on a task measuring working memory capacity, processing speed, inhibition, and in a subsample on response- and attention control. Moreover, boys were found to perform superior to girls on tasks measuring processing speed. However, girls were found to perform better on tasks related to attention and self-control. The results are discussed in relation to the reciprocal association between cognition and schooling and whether these results reflect quality differences between schools in the four Nordic countries; most notably in comparison to Finland.

The starting point for the present study is a research study titled “Prospects for promoting health and performance by school meals in Nordic countries (ProMeal)” (Waling et al., 2016). The general aim of that study was to determine whether overall healthiness, based on diet and learning conditions can be favored by school lunches in four Nordic countries. The study included more than 800 10–11-year-old pupils from Finland (4th grade), Sweden (4th grade), Norway (5th grade), and Iceland (5th grade). In this study, we also measured basic cognitive abilities such as working memory capacity (WMC), inhibition, processing speed, attention, and self-control. The present study focuses on these cognitive measures, the pupils’ age, and their attending grades. The present study provides important insights on whether one year extra of formal schooling may influence basic cognitive abilities already at 10-11 years of age. Results that are of significance in the perspective of the *reciprocal* relation between cognition and school attainments (e.g., Andersson & Lyxell, 2007; Cliffordson & Gustafsson, 2008; Swanson & Howell, 2001) and the ongoing debate following the recurrent PIRLS^i^ and TIMSS^ii^ evaluations. The Nordic countries are considered homogenous with regard to shared economic and social models of the welfare (Berggren & Trägårdh, 2012). The organization “Save the Children” (https://www.savethechildren.net) ranked in 2015 the Nordic countries at the top five positions on an index related to maternal health issues (e.g., educational and economic status). Although all pupils in the present study were in the same age, they differed with regard to number of formal school years. Swedish and Finnish children start compulsory school the year they turn seven years of age and Norwegian and Icelandic children the year they turn six years of age (“Barn och familj i Norden,” 2016). With regard to organizational prerequisites and learning environments, there are some notable differences. Finnish pupils spend the least amount of compulsory instruction time in primary and lower education compared to Swedish, Norwegian and Icelandic pupils. The average hours per year spent in class in 2013 was as follow: Finland, 632 hours; Sweden, 754 hours; Norway, 748 hours; and Iceland 729 hours. Finland had in 2013 the lowest total public expenditure on primary education as a percentage of total public expenditure (including public subsidies to households for living costs, which are not spent in educational institutions): Finland 2.3%; Sweden 3.3%; Norway 3.8% and Iceland 5.1%. In spite of this difference the average class size in primary schools was in 2014 almost identical for Finland, Sweden, and Iceland (18-19 pupils in each class). No data was available for Norway (OECD, 2016).

## Hypothesis

There is substantial evidence that extra time in school does affect intelligence (e.g., Cliffordson & Gustafsson, 2008; Ritchie, Bates, Der, Starr, & Deary, 2013) and that intelligence is intertwined with the aforementioned cognitive constructs (e.g., Sheppard & Vernon, 2008; Unsworth, Fukuda, Awh, & Vogel, 2014). The Nordic countries are regarded as homogenous with respect to economic and social welfare (Berggren & Trägårdh, 2012), but differ with regard to when children enter formal school (“Barn och familj i Norden,” 2016). We, therefore, expected that Icelandic and Norwegian (5th grade) pupils should outperform Swedish and Finnish (4th grade) pupils of the same age on tasks measuring WMC, processing speed, inhibition, attention, and self-control.

Below we elaborate on the cognitive measures used, their underlying constructs, interrelationships, relations to intelligence and of the relations between schooling and intelligence.

## Background

### Interrelationships and Relations of Working Memory, Inhibition, Processing Speed, Attention and Self-Control With Intelligence

The relationships between working memory and inhibition, processing speed, attention, and self-control have been emphasized in several studies. Among those studies, it is common to describe self-control or self-regulatory behavior as connected to the executive functions of working memory. For instance, Hofmann, Schmeichel, and Baddeley (2012) argue that “executive attention” is one of the “main battlefields of self-regulation”. The relationship between inhibition, attention, and working memory is also emphasized in Kane and Engle’s (2003) model of working memory. They argue for a domain-general executive attention mechanism that is responsible for maintaining information in working memory. Without any distractors, relevant information can be retrieved from long-term memory, but when distractors are present, task-irrelevant information will intrude and slow down one’s response and produce more errors. In the Kane and Engle’s (2003) study, it was shown that conflicts arising from performing habit versus goal-oriented Stroop tasks are more problematic for individuals with low WMC. Hence, individuals with lower working memory span conducted more errors in incongruent trials when congruent trials were made up of 75% or 80% of the Stroop task. The authors argue that this result is similar to what is found during dichotic listening tasks (Conway, Cowan, & Bunting, 2001) and antisaccade tasks (Kane, Bleckley, Conway, & Engle, 2001). Studies of children with attention deficit hyperactivity disorder (ADHD) have shown that the lower WMC that these children often exhibit typically manifests in a lack of inhibitory and attentional control, and that processing speed explains much of this deficit (Karalunas & Huang-Pollock, 2013). The significance of processing speed for WMC can also be seen when perceptual speed is controlled for. In those studies, the explained variance associated with WMC is significantly reduced when the speed component is statistically removed (e.g., Salthouse, 1992, 1996). In addition to the intertwined relations of the aforementioned constructs, it is also well known that these constructs are associated with intelligence. For example, Unsworth et al. (2014) showed that a multifaceted view of working memory including scope of attention, attention control and secondary memory explained the individual differences in working memory and its relation to fluid intelligence. In a review by Sheppard and Vernon (2008) it was concluded that intelligence and processing speed is significantly correlated, with a tendency that more complex speed tasks obtained higher correlations. It was further found that the correlations were stronger with general fluid intelligence than with crystallized intelligence. The literature regarding these constructs is massive. With the intent of providing a basic understanding of these constructs, below we briefly describe each construct separately and the relation between schooling and intelligence.

#### Working Memory

Baddeley’s model of working memory is perhaps the most influential model (e.g., Baddeley, 2000). Baddeley’s model consists of a central executive, which is responsible for the control and regulation of cognitive processes and three separable and interacting subsystems: the phonological loop, the visuo-spatial sketchpad, and the episodic buffer. The phonological loop is responsible for speech-based information, the visuo-spatial sketchpad for visual and spatial information, and the episodic buffer for integrating information from both long-term memory and the various components of working memory. The central executive controls the subsystems and regulates goal-oriented behavior, such as planning, inhibition, shifting and updating (Miyake, 2000). The sub-component *inhibition* is responsible for selecting a correct response in the presence of a stronger competing source. *Shifting* is responsible for one’s ability to shift between mental sets, while *updating* is responsible for negotiating new information in relation to old information. Working memory capacity refers to one’s ability to store as much information as possible while simultaneously processing information and is commonly measured using complex working memory tasks (e.g., Daneman & Carpenter, 1980; Redick et al., 2012; Unsworth, Heitz, Schrock, & Engle, 2005).

#### Inhibition

Inhibition is as pointed out often seen as part of the executive control (Miyake, 2000), but can also be viewed as a separate construct, denoted as cognitive inhibition or just inhibition (MacLeod & MacDonald, 2000; but see Dempster & Corkill, 1999, for a different view). MacLeod (2007) defined cognitive inhibition as “…the stopping or overriding of a mental process, in whole or in part, with or without intention” (p. 5). Cognitive inhibition is a mental process and is involved in tasks such as having to inhibit irrelevant information, which can be viewed as different from inhibition that manifests in motor-related behavior, such as inhibiting a motor response. However, several studies have found overlapping brain areas that are involved in both cognitive and motor inhibition. These studies are supported by findings showing that psychiatric patients are usually impaired in tasks assessing both motor response inhibition and cognitive inhibition (see Bari & Robbins, 2013, for a review). In the present study, inhibition is thus regarded as a construct that includes both cognitive and motor inhibition. A general finding among studies is that inhibition plays an important role in development; however, whether the development is linear or protracted is subject for debate (e.g., McAuley & White, 2011).

#### Processing Speed

Processing speed refers to the amount of time it takes to carry out simple or automatic cognitive tasks. It is regarded as a characteristic that is different in each individual and is related to other cognitive abilities. Salthouse (1996) offered two mechanisms for understanding how processing speed is related to changes in cognitive functioning: the limited time and simultaneity mechanisms. The limited time mechanism is associated with one’s ability to complete cognitive operations within a given amount of time. The simultaneity mechanism is associated with one’s ability to maintain information from previous processes by the time subsequent processing is completed. It is well established that processing speed is attributed to the changes in cognitive abilities across childhood (Kail, 1991; Kail, 2007) and adulthood (Salthouse, 1996). Across adulthood, these changes indicate that the gradual age-related decline of more advanced cognitive abilities (e.g., reasoning) is caused by an age-related decline in simple perceptual and cognitive processing speeds (Salthouse, 1992, 1996; Schretlen et al., 2000). The development of processing speed and its significance for other cognitive functions among children was described by Fry and Hale (1996) as a “cognitive developmental cascade.” That is, a sequence of stages in which the current stage affects the forthcoming stages: increasing chronological age >> processing >> speed >> WMC >> reasoning ability. This is a sequence that Nettelbeck and Burns (2010) interpreted as “maturing brain structures improve processing speed, with concurrent improvement in working memory and other cognitive functions” (p. 380).

#### Attention and Self-Control

Attention and self-control are two constructs that share variance. Hence, attention can be regarded as a key component of self-control, and the role of attention is to select information from an array of environmental information (Knudsen, 2007). Self-control is compared to the broader concept self-regulation, which is commonly used to define the self-regulatory processes aimed at controlling emotions, thus overriding pre-potent impulses in relation to external demands (e.g., Hofmann et al., 2012). When a goal is set, top-down attention can modulate more bottom-up attentional inputs, thus inhibiting irrelevant information and preserving the goal of working memory (Kane et al., 2001; Knudsen, 2007). Longitudinal studies have found evidence that self-control is predictive of academic attainments and prosocial behavior (Duckworth & Gross, 2014), as well as intelligence (Moffitt et al., 2011).

### The Relation Between Formal Schooling and Intelligence

In a seminal study, Ceci (1991) provided evidence for a quantitative relationship between schooling and general intelligence. Hence, the number of years of schooling was associated with increasing IQ scores. The perhaps strongest evidence came from “natural” experiments indicating that IQ increased between 2-6 points for every year in school. Among other, Brinch and Galloway (2012) showed that a mandatory attendance reform that was launched in Norway in the 1960s, but differently enforced depending on municipalities, generated variation in an ability test undertaken by all 19 years old as part of their military service. This “natural” experiment allowed for an estimate of the effects of compulsory education. An effect of 3.7 increased IQ per school year was found. Type of schooling and its influence on cognition has also been investigated. When comparing a mandatory enlistment test battery, Cliffordson and Gustafsson (2008) found that the average effect of schooling on cognition was a 2.7 point increase in IQ for each year. On a population level, using data from every individual enlisted in the military in Sweden between 1980 and 1994, Carlsson, Dahl, Öckert, and Rooth (2015) showed that an extra 10 days of schooling increased crystallized intelligence by 1% of a standard deviation, but that measures of fluid intelligence did not. In recent years, a longitudinal study of participants born in 1921 revealed that each year of education had increased the individuals’ IQ score by an average of 0.66 points at age 79 years when controlling for IQ at age 11 years (Ritchie et al., 2013). Although the effects of education on processing speed were small or non-existent, Ritchie et al. (2013) raised the question of whether a difference in education early in life (before the age of 11 years) may also increase one’s processing speed.

In addition to the numerous behavioral studies that have been performed in this area, there are also neuroimaging studies that show how some years, or even days, in school, make a difference with regard to academic performance and how the brain processes information. In a study of pupils in the 2nd and 3rd grades (7-9 years old), Rosenberg-Lee, Barth, and Menon (2011) found evidence that even a single year of schooling exhibited altered brain functions and connectivity, indexed by arithmetic progress. Effects that were most notable included increased activation in the dorsolateral prefrontal cortex (dlPFC) and deactivation in the ventral medial prefrontal cortex. Third-graders also showed a greater connectivity between the dlPFC and a number of posterior brain regions.

## Methods

Using computer- and Web-based tasks, we measured WMC, inhibition, processing speed, and (on a subsample) attention and self-control.

### Participants

A total of 837 10-11-year-old pupils attending the 4th or 5th grade participated in the *ProMeal* study (for more details see Waling et al*.,* 2016). Each country included between 201 and 225 pupils; 48% of the students were boys (Table 1). Of those 837 pupils, 104 did not complete the cognitive tests and/or were excluded from the analyses because they had a diagnosis such as Tourette syndrome, ADHD/attention deficit disorder (ADD), dyslexia, autism, or a combination thereof. In the analyses of processing speed and inhibition, 733 participants were included. For the task that measured WMC, 40 pupils scored zero; they probably misunderstood the task and were thus also excluded from the analysis. Therefore, a remaining sample of 693 pupils was included for the complex working memory task.

**Table 1:** Number of Participating Schools, Classes, and Pupils in the ProMeal Study

	Grade	Schools (*n*)	Classes (*n*)	Pupils (*n*)	Boys *n* (%)	Girls *n* (%)
Total		30	62	837	403 (48)	434 (52)
Finland	4th	9	18	206	99 (48)	107 (52)
Sweden	4th	9	14	197	98 (50)	99 (50)
Norway	5th	6	17	210	92 (44)	118 (56)
Iceland	5th	6	13	224	114 (51)	110 (49)

In a subsample of pupils, we also measured attention and self-control. For these measures, we randomly selected pupils from each school (a total of 212 students). Out of those 212 pupils, we excluded an additional 18 participants, as they only had partial data registered and two participants with a diagnose. Note that there were more Finnish pupils doing this test. The final sample used in the analyses of attention- and response control consisted of 194 participants. It had of course been desirable to collect this data from all participants. However, due to license demands and restricted available research time due to the duration of the school day, the collection of attention and self-control data was restricted to a subsample.

The samples were relatively homogeneous from a socioeconomic perspective; data from a parental questionnaire showed that the children’s parents were highly educated, employed, and faced few economic challenges (Waling et al., 2016). This homogeneity was relatively equal across countries, but there were some differences worth noting. For instance, fewer Finnish parents had a university degree; the percentages of university degrees held for the parents/caregivers in each country were as follows: Finland (51%), Sweden (66%), Norway (72%) and Iceland (58%). Proportions based on a response rate of 80%, 90%, 68% and 97%, respectively. These percentages are higher than the official statistics from the Organisation for Economic Co-operation and Development (OECD) (42%, 39%, 42%, 38%, respectively for tertiary education in 2015) with regard to educational attainments of 24-64 years old (OECD, 2016). However, in the present study, the most of the pupils’ parents/caregivers lived in or in close proximity to university cities and were most likely within the age span of 30-45 years (considering that they had a child between 10-11 years of age). These circumstances probably inflate the percentages compared to the country averages. Note also the low response rate for Norwegian parents.

The percentage of non-native pupils differed to a lesser extent: Finland, 2% (*n* = 3); Sweden, 5% (*n* = 6); Iceland, 7% (*n* = 13); no data were available from Norway. Response rate for Finland, Sweden, and Iceland, were 90%, 80%, 99%, respectively.

### Measures of Cognitive Abilities

*Working Memory Capacity*. The measures of WMC were conducted using a complex working memory task (Unsworth et al., 2005), denoted as child operation (CO)-span, in which a series of interleaved letters and a concurrent arithmetic tasks were presented. The participants had to perform various mathematical operations (addition with the sum of integers always in the range of 3-9) while simultaneously retaining letters in their short-term memory. The scoring process uses the same principle as that of the Wechsler Intelligence Scale for Children (WISC-IV) (Wechsler, 2003) digit span task: the total number of letters to be recalled. The task began at level two with two sequences; the student then proceeded to the next level as long as two sets in a row were answered correctly. Thus, there was no predefined highest level, but the span score ranged from zero to the individual’s highest level (Nyroos, Jonsson, Korhonen, & Eklöf, 2015).

*Inhibition and Processing Speed*: For the measures of inhibition and processing speed, a computer-based Stroop task was used. The pupils were shown a sequence of words, one at a time, that was either congruent, incongruent, or neutral with their coloring and the students were required to indicate the color of the letters (MacLeod & MacDonald, 2000). The words were presented in each student’s respective language. Four blocks of 32 trials with a total of 128 trials were presented. Each individual block consisted of 8 neutral trials, 12 congruent trials, and 12 incongruent trials. These blocks were presented in a random order. Blocks containing congruent and incongruent trials were comprised of color words (black, blue, red, and yellow). The neutral block (e.g., “car” printed in black) consisted of nouns of similar lengths as the color words, presented in colored letters. These blocks are denoted as conditions. Three measures were extracted: the reaction time (RT) in milliseconds for the incongruent, congruent, and neutral conditions. The RTs for congruent and neutral conditions were used as the dependent measure for processing speed (the shorter the RT, the faster the processing speed). In the Stroop paradigm, congruent and neutral words can be used as measures of processing speed, as they do not elicit any conflict or inhibition (e.g., Tam, 2013).

The RT for incongruent conditions denoted as “Stroop inhibition,” can be seen as indicative of inhibition; when comparing incongruent with congruent conditions, the RT is typically higher for the incongruent condition (i.e., it represents a Stroop inhibition effect).

*Attention and Self-Control:* For attention and self-control, we used the Integrated Visual and Auditory Continuous Performance Test (IVA+Plus). IVA+Plus is a computer-based test of attention and response control (Sandford & Turner, 2004a). In the IVA+Plus, two stimuli are presented simultaneously (the numbers “1” and “2”). There are 250 trials in each modality presented either on the computer screen or in headphones. The participants are required to respond only to target “1” and to inhibit their response to target “2”, irrespective of the presented modality. The frequencies of “1” and “2” vary across tests, being common or rare, with increasing risk of commission and omission errors, respectively.

The IVA+Plus full-scale response quotient is a measure of an individual’s overall ability to make accurate responses. The scale also includes both visual and auditory modalities and is built upon separate scores of vigilance (measure of inattention, derived from two types of commission errors), focus (total variability of processing speed, derived from the variability of correct responses), and speed (attention processing problems in relation to slow discriminatory mental processing, derived from RTs for correct responses). The full-scale attention quotient was used as a proxy of attention in the present study.

The IVA+Plus full scale response quotient is a measure of an individual’s ability to regulate his or her responses and to respond appropriately; in the present study, this scale was used as a proxy of self-control. The scale includes both visual and auditory modalities and is built upon separate scores of prudency (measures of impulsivity and response inhibition, derived from omission errors), consistency (the ability to stay on task, derived from the general reliability and variability of RTs), and stamina (identifies problems with sustaining attention and maintaining effort, derived from the RTs when making correct responses during the first and last 200 trials). The full-scale response quotient was used as a proxy of self-control. Higher scores are always indicative of better attention and self-control, respectively (“Scoring the IVA+Plus,” 2016).

### Procedure

#### Data Collection

The data were collected between October 2013 and May 2014. The WMC task and tests of inhibition and processing speed were performed with all participating pupils during the morning lecture. One week later, a continuous performance test measuring attention and self-control was administered the sub-sample, about two hours after lunch on three separate days. The procedure for administering the CPT task was identical for all participants. However, for the purposes of the present study, only the values from day one were used in the analyses. To facilitate comparisons, the entire data collection process was completed via computer; hence, the same instruments were used and the tasks were therefore presented in the same structured way in all countries. Before starting each test, the same instructions were given to all pupils. Data collection for the CO-span and Stroop task was in Sweden, Finland and Norway conducted in groups of 5-10 pupils. On Iceland, special computer rooms with space for 20 pupils were used. For the IVA+Plus measure the data collection was in all countries conducted in groups of 3-5 pupils. The psychometric properties of each dependent variable is described below.

*CO-Span.* The analyses of skewness and kurtosis revealed no problems with either. Skewness was -0.90 (*SE* = 0.90) and kurtosis 0.640 (*SE* = 0.18). The dependent variable for WMC was the number of correct recalled letters. Higher values were interpreted as better WMC. To control for that effort was allocated to the concurrent math tasks, the dependent variable “percentages math correct” was also analyzed. For percentages math correct, the skewness was -1.62 (*SE* = 0.09) and kurtosis 4.60 (*SE* = 0.18); they were, therefore, log transformed. Following the transformation, the mean values were -0.69 (*SE* = 0.09) and -0.82 (*SE* = 0.18) for skewness and kurtosis, respectively. The analysis of percentages math correct was conducted on the transformed values.

*Stroop*. RT was measured in milliseconds and aggregated to the level of mean values for each pupil and condition. Initial analyses revealed relative high skewness and kurtosis among the dependent variables. The skewness for the neutral, congruent, and incongruent conditions were between 2.07 and 3.38 (*SE* = 0.09) and kurtosis between 9.24 and 27.30 (*SE* = 0.18). After the log transformation, the mean values for skewness ranged from 0.56-0.69 (*SE* = 0.09) and the mean value for kurtoses were between 0.69 and 1.34 (*SE* = 0.18). The analyses were conducted on the transformed values.

*Attention and Self-Control.* The skewness and kurtosis analyses revealed that there were no problems with either. Skewness for the full-scale attention quotient was -0.70 (*SE* = 0.17) and 0.02 (*SE* = 0.34) for kurtosis. The corresponding values for the full-scale response quotient were -0.74 (*SE* = 0.17) and 0.64 (*SE* = 0.34), respectively. The IVA+Plus full-scale attention quotient and the full-scale response quotient were used as the dependent variables. Higher scores were interpreted as better attention and self-control.

### Factor Structure and Factorial Invariance

In order to assess whether the same constructs were measured across countries and gender initial principal component analyses (PCA) extracted the factor structure for all participants with regard to the tasks that all participants performed (WMC, math task, Stroop neutral, congruent, incongruent). The PCA analyses (SPSS) on the five dependent variables was conducted with oblimin rotation. This was followed by a factorial invariance analyses which evaluated whether the same factor structure- and loading were present across countries and gender. Factorial invariance was estimated through both configural and metric invariance using AMOS. The model fit for configural invariance (number of factors across countries and gender) was obtained by using the comparative fit index (CFI; Bentler, 1990; Hu & Bentler, 1999), the Tucker and Lewis index (TLI; Tucker & Lewis, 1973), and the root-mean-square error of approximation (RMSEA; Browne & Cudeck, 1992). According to Bentler (1990), CFI and TLI values greater than .90 are indicative of an acceptable fit. RMSEA-values below .05 represent a good fit (Byrne & Campbell, 1999). To evaluate metric invariance (whether the same factor loadings were present across countries and gender) a model comparison between a fully constraint model and an unconstraint model was conducted.

The Kaiser-Meyer-Olkin (KMO) measured verified that the samples were adequate for factor analyses (.77) and that Bartlett test was significant. A KMO value above .6 is considered to be a minimum, and a significant result (*p* < .05) on Bartlett´s test is needed for data to be suitable for factor analysis (Field, 2013). The PCA extracted two factors with an eigenvalue above 1 explaining 75.13% of the variation was extracted. Table 2 shows the rotated factor loading.

**Table 2:** Factor Loading for Exploratory Factor Analysis With Oblimin Rotation

Dependent variables	Factor 1	Factor 2
Stroop Congruent	.94	
Stroop Incongruent	.94	
Stroop Neutral	.93	
Math percent correct		.78
CO-span		.75

*Note.* Factor loadings below .30 are suppressed.

The analysis of configural invariance across countries and gender displayed a CFI of .999, TLI of .998 and RMSEA of .08. The analyses of metric invariance showed that chi-square difference was nonsignificant, *p* = .27. Altogether, indicating a good fit with regard to both configural and metric invariance across countries and gender.

These analyses were not conducted on measures of attention and self-control. Those measures were, as pointed out, obtained on a subsample and therefore too small to be used in an evaluation of factor structure.

### Ethics

Written informed consent was obtained from all participating parents/caregivers before the pupils entered the ProMeal-study. In Finland, informed consent was also collected from the pupils. In all countries, the pupils were able to deny participation even if parents/caregivers had consented participation. The study was conducted according to the guidelines laid down in the Helsinki Declaration of 1975, as revised in 2008, and all procedures involving human subjects were approved by the Ethical Committee of the University of Turku in Finland, The National Bioethics Committee (56363); The Icelandic Data Protection Authority (VSN- 13-088) in Iceland; The Data Protection Official for Research in Norway; and The Regional Research Ethics Review Board, the Faculty of Medicine, Umeå University, in Sweden (2013-212-31O).

### Statistical Analyses

Since the sample size differed depending on the task, and in order to assess the effects associated with the specific aforementioned constructs, we ran separate analyses of variance (ANOVA) with countries and gender serving as between-subjects variables, while the tasks targeting each construct separately served as the dependent variables. For Each ANOVA, partial eta square (η^2^_p_) was reported as a measure of effect size. The omnibus tests of each dependent variable were followed by Bonferroni’s adjusted post hoc tests at a .05 level

## Results

The results comparing countries are presented according to the performed tasks, CO-span, Stroop (congruent, incongruent and neutral), and IVA+Plus (attention and response control) and interpreted in terms of corresponding constructs. Table 3, 4 and 5 show the mean values and standard deviations for all the dependent variables and are based on non-transformed values. Included are also the mean value range across individual schools. Initial T-tests comparing parents’ level of education (university degree with no university degree) revealed no effect of education on any of the dependent measures, all *p* > .05.

*CO-Span:* An ANOVA with countries and gender serving as the between-subjects factors and CO-span scores acting as the dependent variable revealed a significant effect of country, *F*(3,685) = 9.4 *p* < .001, η^2^_p_ = 0.04. However, no effect of gender, *F*(1,685) = 0.71, *p* = .40, η^2^_p_ = 0.001, and no interaction effect between gender and country, *F*(3,685) = 1.13, *p* = .34, η^2^_p_ = 0.005, were found. The post hoc tests confirmed that Finnish pupils outperformed pupils from all other countries. No other comparisons differed significantly (Table 3). The analysis shows that Finnish pupils as a group scored higher than pupils from all other countries on a measure of WMC.

**Table 3:** Average Measures and Standard Deviations of CO-Span and Math Tasks Across Countries, Gender and Range Measures

Group	*n*	Task (correct response in ms)					
		CO-Span			CO-Span Math (percent)		
		*M*	*SD*	Range	*M*	*SD*	Range
Finland							
Boys	83	4.40	1.08		90.18	10.03	
Girls	104	4.32	1.16		89.38	8.75	
Total	187	4.35	1.12	3.94-4.58	89.74	9.33	87.19-92.46
Sweden							
Boys	71	3.72	1.16		91.42	7.12	
Girls	77	3.73	1.15		89.96	9.92	
Total	148	3.72	1.15	3.6-4.10	90.66	8.69	87.47-94.32
Norway							
Boys	87	4.17	1.14		89.26	8.04	
Girls	107	3.86	1.14		89.53	9.72	
Total	194	4.00	1.25	3.73-4.42	89.41	8.98	85.95-91.49
Iceland							
Boys	82	3.90	0.93		90.20	8.12	
Girls	82	4.00	1.06		90.21	8.93	
Total	164	3.95	1.00	3.57-4.34	90.21	8.51	88.74-91.53

*Note.* CO-span = Child operation span task, a measure of complex working memory. CO-span math = operation span, the concurrent math task in percent correct response. Range = mean value range across individual schools.

With regard to the concurrent arithmetic tasks, there were no differences between countries, *F*(3,685) = 0.65, *p* = .58, η^2^_p_ = 0.003, or gender, *F*(1,685) = 0.52, *p* = .47, η^2^_p_ = 0.001, nor were there any interaction effects between gender and country, *F*(3,685) = 0.32, *p* = .81, η^2^_p_ = 0.001. Only 3.5 percent of the participants scored below 70% math correct, and those were evenly distributed across countries and gender. Hence, the cognitive efforts spent by the pupils during the concurrent tasks were equal across all four countries and for both genders.

*Stroop:* The mean RTs for neutral, congruent and incongruent show that Icelandic and Finnish pupils responded faster than Norwegian and Swedish pupils; boys were faster than girls. Separate ANOVAs with countries and gender as the between-subjects factors revealed main effects of countries for neutral words, *F*(3,724) = 13.30, *p* < .001, η^2^_p_ = 0.5, and congruent words, *F*(3,724) = 15.76, *p* < .001, η^2^_p_ = 0.06. The effect of gender was also significant across neutral and congruent conditions, with shorter RTs for boys, as well as for neutral, *F*(1,724) = 8.14, *p* = .004, η^2^_p_ = 0.01, and congruent words, *F*(1,724) = 11.15, *p* = .001, η^2^_p_ = 0.02. However, there was no country × gender interaction for neutral or congruent conditions, *F*(3,724) = 2.00, *p* = .11, η^2^_p_ = 0.008, *F*(3,724) = 0.65, *p* = .58, η^2^_p_ = 0.003, respectively. Post hoc analyses confirmed that Finnish and Icelandic pupils had significantly shorter RTs than Swedish and Norwegian pupils. No other comparisons were significant (Table 4). These response differences indicate higher processing speed fore Icelandic and Finnish pupils compared to Norwegian and Swedish pupils and higher processing speed for boys compared to girls.

**Table 4:** Average Measures With Standard Deviations (Milliseconds) of Stroop Tasks Across Countries, Gender and Range Measures

Group	*n*	Task (reaction time)								
			Stroop Neutral			Stroop Congruent			Stroop In-Congruent		
		*M*	*SD*	Range	*M*	*SD*	Range	*M*	*SD*	Range
Finland										
Boys	86	1379	474		1255	326		1399	430	
Girls	105	1488	419		1368	331		1565	440	
Total	191	1439	446	1227-1527	1317	332	1113-1379	1490	442	1348-1645
Sweden										
Boys	78	1627	728		1531	694		1645	826	
Girls	86	1660	868		1567	505		1692	562	
Total	164	1664	802	1282-1921	1550	601	1221-1737	1669	698	1270-2030
Norway										
Boys	90	1484	522		1439	484		1504	503	
Girls	110	1713	525		1609	532		1748	571	
Total	200	1610	534	1473-1807	1532	516	1358-1735	1638	554	1440-1888
Iceland										
Boys	88	1338	439		1262	376		1344	399	
Girls	90	1336	496		1336	498		1433	482	
Total	178	1350	468	1196-1720	1299	443	1116-1640	1398	444	1196-1806

*Note.* Stroop neutral = measure of processing speed. Stroop, congruent = measure of processing speed. Stroop incongruent = measure of inhibition. Range = mean value range across individual schools.

For the incongruent conditions of the Stroop task, there was a significant effect of country, *F*(3,724) = 11.80, *p* < .001, η^2^_p_ = 0.05, and gender, *F*(1,724) = 17.92, *p* < .001, η^2^_p_ = 0.02, with boys responding faster than girls. However, there was no country × gender interaction, F(3,724) = 0.99, *p* = .40, η^2^_p_ = 0.004. Post hoc analysis confirmed that Icelandic and Finnish pupils had significantly shorter RTs than Swedish and Norwegian pupils. No other comparisons were significant. The shorter response time indicates a better ability to inhibit a prepotent response among Icelandic and Finnish pupils compared to Swedish and Norwegian pupils and that boys were better than girls.

*IVA+Plus:* There was no main effect of country for the IVA+Plus full-scale attention quotient, *F*(3,186) = 1.91, *p* = .13, η^2^_p_ = 0.03. The analysis of gender revealed a significant effect, *F*(1,186) = 4.72, *p* = .03, η^2^_p_ = 0.03, with girls outperforming boys. No country × gender interactions was found, *F*(3,186) = 1.20, *p* = .31, η^2^_p_ = 0.02.

The analysis of the IVA+Plus full-scale response-control attention quotients revealed a tendency of significant effects of country, *F*(3,186) = 2.61, *p* = .05, η^2^_p_ = 0.04, with pupils from Finland and Iceland outperforming pupils from Sweden and Norway. The analysis of gender was significant, *F*(1,186) = 9.70, *p* = .002, η^2^_p_ = 0.05, with girls outperforming boys. However, no country × gender interactions was found, *F*(3,186) = 0.55, *p* = .65, η^2^_p_ = 0.009 (Table 5). These results indicate that that Finnish and Icelandic pupils had better self-control than Swedish and Norwegian pupils and that girls’ self-control and attention was better than boys’, irrespectively of country.

**Table 5:** Average Measures With Standard Deviations of IVA+Plus Across Countries and Gender

Group	*n*	Task (correct response)					
		IVA+Plus; attention			IVA+Plus; response		
		*M*	*SD*	Range	*M*	*SD*	Range
Finland							
Boys	39	83.54	20.45		82.85	20.47	
Girls	45	96.17	16.17		89.89	18.07	
Total	84	90.38	19.22	84.00-98.93	86.62	19.43	76.17-99.14
Sweden							
Boys	18	81.67	22.10		71.28	18.62	
Girls	22	85.23	24.70		81.32	24.18	
Total	40	83.63	23.34	67.00-108.00	76.80	22.17	65.20-94.60
Norway							
Boys	8	83.63	18.71		74.00	24.72	
Girls	19	93.89	20.72		91.05	12.20	
Total	27	90.85	20.36		86.00	18.81	80.60-91.00
Iceland							
Boys	22	93.27	14.73		83.09	21.48	
Girls	21	93.76	19.64		88.00	15.02	
Total	43	93.51	17.10	77.75-106.75	86.49	18.56	69.50-90.40

*Note.* IVA+Plus attention = measure of attention control. IVA+Plus response = measure of response control. Range = mean value range across individual schools.

Table 6 summarizes the differences between countries and gender. Note that higher CO-span, math tasks, IVA+Plus (full scale- attention and response control) are regarded as better performance. While shorter response time for Stroop tasks (neutral, congruent, incongruent) are regarded as better performance.

**Table 6:** Comparing Countries and Gender

CO-Span (complex working memory task)	Finland > Sweden & Iceland & Norway***
	Boys = Girls
Math Task (concurrent working memory task)	Finland=Sweden=Iceland=Norway
	Boys = Girls
Stroop Neutral (processing speed)	Finland & Iceland > Sweden & Norway***
	Boys > Girls**
Stroop Congruent (processing speed)	Finland & Iceland > Sweden & Norway***
	Boys > Girls**
Stroop Incongruent (inhibition)	Finland & Iceland > Sweden & Norway***
	Boys > Girls**
IVA+Plus Full Scale Attention attention)	Finland=Sweden=Iceland=Norway *ns.*
	Boys < Girls*
IVA+Plus Full Scale Response Control (response control)	Finland & Iceland > Sweden & Norway^†^
	Boys < Girls**

^†^*p* = .05. *p < .05. **p < .01. ***p < .001.

## Discussion

The present study set out to investigate the potential differences between 10-11-year-old pupils from Finland, Sweden, Norway and Iceland, with regard to their basic cognitive capacities. The 10-11-year-old pupils from Iceland and Norway were attending the 5th grade, and the Swedish and Finnish pupils were in 4th grade. We expected that 5th-grade pupils from Norway and Iceland should outperform 4th grade pupils from Finland and Sweden, as they were in the same age range (10-11 years) but had attended school for one year longer; hence a schooling effect based on more schooldays experience. Initial factor analyses indicate that the measures can be considered as invariant across countries and gender and thus valid for the statistical analyses.

The results from the present study showed that Finnish 4th grade pupils never were outperformed on any of the dependent variables. In addition, the analyses of gender differences did reveal that there was no gender-related difference with regard to CO-span. For neutral and congruent Stroop conditions, the boys were faster. However, on the other hand, girls performed better on the IVA+Plus measures. The same pattern was also seen with regard to the IVA+Plus measure of response control.

The between countries differences were unexpected, especially the difference between Finland, Sweden and Norway. Although the results do not rule out socioeconomic factors, it seems unlikely that the obtained differences are caused by socioeconomic differences among the participating countries, considering the homogeneity among the Nordic countries (Berggren & Trägårdh, 2012).

Below each construct is addressed separately and discussed with respect to potential quality differences among schools in the Nordic countries as reflected in the PIRLS and TIMMs evaluations.

*Working Memory Capacity.* In the present study, WMC was measured by the CO-span task, in which participants had to process a mathematical task while simultaneously retaining letters in their short-term memory. The analyses showed that Finnish pupils scored higher than pupils from all other countries. When measuring WMC using complex working memory tasks, it is important that the participants process the concurrent task while remembering the stimuli intended to measure WMC. If the participants choose to ignore the concurrent tasks, the complex working memory tasks turn into a simple span task that only provides a measure of short-term memory capacity. However, only 3.5% of the participants scored below 70% math correctly, and those were evenly distributed across countries and gender. Indicating that they allocated cognitive resources for the concurrent tasks. In addition, there was no difference in performance between countries for the concurrent arithmetic tasks; the average scores were almost exactly the same.

*Processing Speed.* Processing speed was measured using the neutral and congruent conditions of the Stroop task. Overall, it was found that Finnish 4th and Icelandic 5th grade pupils had shorter RTs than Norwegian 5th and Swedish 4th grade pupils. These results indicate that Finnish and Icelandic pupils processed information faster.

*Inhibition.* In a Stroop incongruent condition, the task is to name the ink color when the word does not correspond to that color (the word “red” printed in “blue”). The prepotent response is to read the word (i.e., red). Hence, to be successful, the pupil has to inhibit the prepotent response to read the word and instead select the color “blue”, a so-called incongruent Stroop task. This is a well-known phenomenon and is frequently used as a measure of inhibition. The results indicate that the differences seen in the incongruent conditions reflect differences in one’s ability to inhibit, as Finnish 4th grade and Icelandic 5th grade pupils outperformed Swedish (4th grade) and Norwegian (5th grade) pupils.

*Attention and Self-Regulation.* The IVA+Plus is a highly demanding (mind-numbing) task; in the present study, it was used to extract measures of attention and self-control. The full-scale attention control quotation was viewed as a measure of one’s overall ability to make a correct response. The full-scale response control quotation scale was viewed as a measure of an individual’s ability to regulate his or her responses, thus enabling him or her to respond appropriately. In the present study, these measures were seen as indicative of attention and self-control, respectively. The results showed that Icelandic 5th and Finnish 4th grade pupils outperformed Swedish 4th and Norwegian 5th grade pupils with regard to self-control. There was no main effect for attention.

Although self-control is not a“pure” cognitive task, it is closely related to the constructs of working memory and processing speed (Hofmann et al., 2012).

*Gender Differences.* The analyses of gender differences revealed no difference with regard to WMC, which is in line with previous research (Brocki & Bohlin, 2004). However, for speed of information processing (Stroop neutral and congruent), the boys were faster – a result that could indicate that boys processed information faster than girls. It is also possible that the effect of processing speed is associated with one’s reading/decoding ability. However, in general, girls are better at tasks involving reading (Mullis, Martin, Foy, & Drucker, 2012), and decoding skills are usually mastered around the 4th grade for both boys and girls (Clinton et al., 2014). An alternative explanation for this difference is that boys have faster RTs on computer-based tasks and not on the speed of information processing per se. In fact, when using a computer-based task, boys have been found to be faster than girls (see Roivainen, 2011, for a review).

The results, which indicated that girls had better response and attention control than boys, could be related to attention ability and the ability to engage in self-control; this may be a function of maturity. However, it could also be attributed to the finding that boys respond faster and thus produce more commission errors when being administered the IVA+Plus test (Sandford & Turner, 2004a).

We did expect that one year extra in school would have some effect on cognition and therefore differentiate 5th grades from 4th grade pupils, hence providing additional support for the hypothesis of schooling effects. To our surprise pupils from Finland, attending 4th grade was not, on any measure, outperformed by pupils attending 5th grade (Iceland & Norway).

It is possible that the differences found between these Nordic countries originate in differences in cognition established already when pupils begin school. However, as pointed out above, if considering socioeconomic indicators such as parents’ educational level, the results found in the present study does not support such an interpretation (especially the difference between Finnish, Swedish and Norwegian pupils). In addition, fewer parents of Finnish pupils had a university degree compared to Swedish and Norwegian parents.

A potential explanation is, of course, the time in school and the economic spending on schools in each country. However, as pointed out above, Finnish pupils spent the least amount of compulsory instruction time in primary and lower education and has in relation to Sweden, Norway and Iceland the lowest public expenditure on primary education (OECD, 2016).

Could it be that the results reflect quality differences in the schooling offered in the different Nordic countries? Most notably between Finland and the other Nordic countries. It is well known that the constructs investigated in the present study are closely related to and predictive of demanding subjects such as reading, math, and science. Working memory is perhaps the most frequently investigated cognitive construct in that respect. The evidence of WMC with regard to performance on basic school subjects such as reading (Swanson, 2003; Swanson & Howell, 2001), mathematics (Andersson & Lyxell, 2007; Clark et al., 2014), learning to spell (Ormrod & Cochran, 1988), and reasoning (Kyllonen & Christal, 1990) is extensive. The progress of children’s WMC, including executive function, also supports the development of self-control and, ultimately, self-regulation. Hence, children become increasingly self-directed, advancing their ability to achieve goals autonomously as a function of a developing working memory. In addition, self-control predicts academic performance over and above intelligence (Duckworth, Quinn, & Tsukayama, 2012).

With this in mind it is interesting to discuss the results in relation to the Progress in PIRLS and TIMSS evaluations from the last few years (see Mullis, Martin, Foy, & Arora, 2012 and Mullis, Martin, Foy, & Drucker, 2012, for an overview). One emphasis in the discussions following the PIRLS and TIMSS reports has been on the performance on reading and math among Swedish and Norwegian 4th-grade pupils, especially in comparisons to Finnish pupils (Iceland did not participate in PIRLS and TIMMS). Given the assumption that the differences found in PIRLS and TIMMS are valid and are not caused by other parameters, such as motivational factors (Eklöf, 2007), a question to ask is whether the effects in the present study reflect the differences found in the PIRLS and TIMSS evaluations of 4th-grade pupils. Schooling effects that are not based on more years in school, but on quality differences between countries that are manifested in the PIRLS and TIMSS evaluations and lead to developmental differences in cognition by the 4th grade. This reasoning is of course highly speculative, as we did not have any data from PIRLS or TIMMS that could be used as a comparison. However, this conclusion seems feasible considering studies showing that cognitive training can provide both behavior and brain activation differences within a time frame of only five weeks (Dahlin, Neely, Larsson, Bäckman, & Nyberg, 2008).

*Strengths and Limitations*. The relatively large sample size, the standardized and computerized tasks, trained researchers collecting data and the fact that the tasks were not part of the curriculums has to be considered as strengths. There are, however, some limitations. Although we have data from OECD (OECD, 2016) on between country differences with regard to organizational prerequisites and learning environments, there are probably differences that are not accounted for. Pupil’s compliance when taking the tests is another potential limitation. It is possible that Finnish pupils were more compliant in taking the test, simply doing as they were instructed without fuss. Conversely, being compliant with instructions can also reflect one’s ability to maintain focus, and this might thus be indicative of attention and self-control abilities. Although, the measures of attention and self-control were underpowered, the results pointed in the same direction as the other analyses. It is therefore possible that the trend toward significance with respect to the analyses of response control underestimated the real effect; hence, there may be a risk for a type II error. The present study was not longitudinal, nor did we measure the pupils when they entered school. To investigate this, a longitudinal design measuring cognitive functioning across time is needed, and these findings should be measured against one’s progress in math, science, and reading; however, this was not within the scope of the present study. Another limitation is that the sampling was conducted in, or in close proximity to, university cities. As a consequence, the proportion of academic parents was relative high, which narrows the external validity. However, it is likely that the differences found in this study would be maintained or even increase in a more heterogeneous sample.

The limitations considered, it is important that future studies focus on quality indicators that might differ between the Nordic countries. These quality indicators should include cognitive demanding tasks such as math, reading and science. They should also include other tasks that facilitate self-control/regulation. In this context, it is worth to mention Blair, Gamson, Thorne, and Baker’s (2005) argument that math education in early school years is a central mechanism through which schooling influences one’s fluid abilities. “Changes in aspects of experience associated with the utilization and repeated practice of prefrontally based fluid cognitive skills that begin relatively early in life are likely to lead to relatively enduring changes in performance on measures of fluid intelligence” (p. 97).

### Conclusions

The results in the present study did not support the hypothesis that an additional year in school will affect basic cognition, though an effect may be present within countries. An interesting finding was that pupils from Finland attending 4th grade were not, on any measure, outperformed by pupils attending 5th grade (Iceland & Norway). Interesting aspects to pursue in future studies are to investigate whether there are cognitive differences among children in these four Nordic countries already at the beginning of formal school and longitudinally investigate whether there are specific quality indicators in school that subsequently affect pupils’ cognitive abilities. A reasonable starting point should be to evaluate those cognitive abilities known to be predictive of school attainments, such as math and reading, as well as to assess the teaching and pedagogical approaches associated with those subjects.
